# Molecular characterization of two novel echovirus 18 recombinants associated with hand-foot-mouth disease

**DOI:** 10.1038/s41598-017-09038-y

**Published:** 2017-08-16

**Authors:** Haihao Zhang, Yilin Zhao, Hongbo Liu, Hao Sun, Xiaoqin Huang, Zhaoqing Yang, Shaohui Ma

**Affiliations:** 1 Institute of Medical Biology, Chinese Academy of Medical Sciences, and Peking Union Medical College, Kunming, 650118 PR China; 2 Yunnan Key Laboratory of Vaccine Research Development on Severe Infectious Disease, Kunming, 650118 PR China

## Abstract

Human echovirus 18 (E-18) is a member of the enterovirus B species. To date, sixteen full-length genome sequences of E-18 are available in the GenBank database. In this study, we describe the complete genomic characterization of two E-18 strains isolated in Yunnan, China. Pairwise comparisons of the nucleotide sequences and the deduced amino acid sequences revealed that the two Yunnan E-18 strains had 87.5% nucleotide identity and 96.3–96.5% amino acid identity with the Chinese strain. Phylogenetic and bootscanning analyses revealed the two E-18 strains had the highest identity with other several EV-B serotypes than the other E-18 strains in the *P*3 coding region, especially, 3*B* region of the Swine Vesicular disease virus (SVDV) strain HK70, indicated that frequent intertypic recombination might have occurred in the two Yunnan strains. This study contributes the complete genome sequences of E-18 to the GenBank database and provides valuable information on the molecular epidemiology of E-18 in China.

## Introduction

The genus *Enterovirus*, within family *Picornaviridae*, order *Picornavirales*, can be divided into 13 species: enterovirus A–J, and rhinovirus A–C (www.picornaviridae.com), comprising more than 100 serotypes. Enterovirus B (EV-B) containing 63 serotypes consists coxsackieviruses B (CV-B1-6), CV-A9, echoviruses (E-1–7, 9, 11–21, 24–27, 29–33) and enteroviruses named with digital serial numbers (EV-B69, 73-75, 77-88, 93, 97-101, 106-107,110-113) and the simian enterovirus SA5 (www.picornaviridae.com/enterovirus/ev-b/ev-b.htm). EVs are small, single-stranded, non-enveloped and positive-sense RNA viruses. The EV genome consisting of approximately 7,400 nucleotides (nt), contains a long open reading frame (ORF) flanked by a 5′-untranslated region (UTR) and a 3′-UTR^1, 2^. The 5′-UTR of EVs has an internal ribosomal entry site that drives translation initiation. The 3′-UTR, containing a long poly (A) stretch, is very important in RNA replication^3, 4^. The ORF encodes a polyprotein that can be processed into three polyprotein precursors: *P1*, *P*2, and *P3*. *P1* encodes *VP1–4* structural proteins. *P*2 and *P3* encode *2A–C* and *3A–D* non-structural proteins, respectively^5^.

EVs infect about a billion people annually worldwide. Though the majority of clinical symptoms are asymptomatic or mild, such as fever, irritation, agitation, sore throat, headache, myalgia, vomiting, diarrhoea, hand-foot-and-mouth disease (HFMD) and others, a few patients may develop severe neurological syndromes as aseptic meningitis, acute flaccid paralysis (AFP), hypertensive heart failure (HHF), dilated cardiomyopathy and death^5^. Of these symptoms, the viruses of species EV-B are the most common viral cause of aseptic meningitis^6–8^.

Echovirus (E-18) belongs to species EV-B. The prototype strain (Metcalf) of E-18 was isolated in 1995 from a patient with diarrhoea^9^. Outbreaks of aseptic meningitis caused by E-18 have been frequently reported^10–16^. To date, except for the prototype strain, there are fifteen E-18 full-length genome sequences in the GenBank database, isolated from the patients with meningitis or contact persons, respectively. In this study, we determined the full-length genome sequences of two E-18 strains (strain A83/YN/CHN/2016, and strain A86/YN/CHN/2016), isolated in the Yunnan province of China in 2016 from two children with HFMD aged 5 and 4.2 years, respectively. This is the first report of E-18 and its probable association with HFMD.

## Results

### Molecular serotyping and primary characterization

The isolates A83/YN/CHN/2016 and A86/YN/CHN/2016 (abbreviated as A83 and A86) were recovered from human rhabdomyosarcoma (RD) cells. They cannot produce a cytopathic effect in KMB17 human embryonic lung diploid fibroblasts (KMB17) and human lung cancer (A549) cell lines. Partial *VP1* sequencing and molecular typing by the online genotyping tool^17^ showed that the serotype of the two isolates is E-18. In addition, complete *VP1*-coding region alignment revealed the two strains had 79.0–79.2% nt and 92.0–92.7% amino acid identities with E-18 prototype strain Metcalf (AF317694).

### Complete genome analysis

The complete genomes of the two strains consisted of 7,404 and 7,399 nt, respectively. They encoded a polypeptide of 2,192 amino acids. The coding sequences were flanked by a non-coding 5′-UTR of 745 nt and a non-coding 3′-UTR of 94 or 100 nt, respectively. The two Yunnan E-18 strains shared 80.4% similarities in the complete genome and 95.4–95.7% similarities in the deduced amino acid sequence with the prototype strain Metcalf, there were 11-nt and 16-nt deletions at the 3′-end of their genomes, and 5 nt insertions and one nt deletion at the 5′-UTR, respectively. The overall base compositions of the strains A83 and A86 genomes were 28.3% A, 22.8% G, 24.9% C and 23.9% U, and 28.2% A, 22.9% G, 24.9% C and 24.0% U, respectively. Compared with other E18 strains, the nt sequences of non-structural regions were more variable (74.2–91.5%), while, the sequences of structural regions were highly conserved (91.3–98.3%). Strain A83 shared 99.7% nt and 99.5% amino acid identities with A86 strain. A comprehensive comparison of different genomic regions of the nt sequences and deduced amino acid sequences of strains A83 and A86 with the E-18 prototype strain and other E-18 strains are shown in Table 1.

**Table 1:** Nucleotide and amino acid identities between the two Yunnan E-18 strains, Metcalf and other E-18 in all sequenced genomic regions.

Nucleotide identity (%) [Amino acid identity (%)]				
Region	A83/YN/CHN/2016		A86/YN/CHN/2016	
	Metcalf	Other E-18	Metcalf	Other E-18
5′UTR	81.7	85.1–97.7	81.7	84.8–97.0
VP4	80.7(92.8)	85.0–96.6 (95.7–98.6)	78.7(89.9)	83.6–94.7(95.7–100)
VP2	80.5(96.5)	86.0–98.3 (98.5–100)	80.5(96.5)	86.0–98.3(98.5–100)
VP3	79.6(96.2)	87.2–97.6 (96.7–97.9)	79.9(97.1)	87.4–97.8(97.5–98.7)
VP1	79.0(92.0)	86.9–97.2 (94.8–97.9)	79.2(92.7)	87.5–97.7(95.8–99.3)
2A	77.8(93.3)	78.4–90.7 (90.7–96.0)	77.8(93.3)	78.7–90.7(90.7–96.0)
2B	79.1(97.0)	75.1–90.9 (90.9–100)	79.1(97.0)	75.1–90.9(90.9–100)
2C	82.4(97.0)	79.4–91.5 (94.8–97.3)	82.3(96.7)	79.3–91.4(94.5–97.0)
3A	77.2(92.1)	75.7–81.3 (93.3–100)	77.2(92.1)	75.7–81.3(93.3–95.5)
3B	78.7(95.5)	74.2–81.8 (95.5–100)	78.8(95.5)	77.3–81.8(95.5–100)
3C	82.3(97.3)	76.3–81.1 (95.6–97.3)	82.3(97.3)	76.3–81.1(95.6–97.3)
3D	80.2(96.6)	77.5–80.9 (94.3–95.7)	80.2(96.1)	77.5–80.9(94.3–95.7)
3′UTR	85.9	81.0–84.2	85.1	80.0–85.1
Genome	80.4(95.4)	81.8–88.5 (95.8–97.2)	80.4(95.5)	81.8–88.5(95.9–97.3)

### Phylogenetic analysis

**Figure 1:**
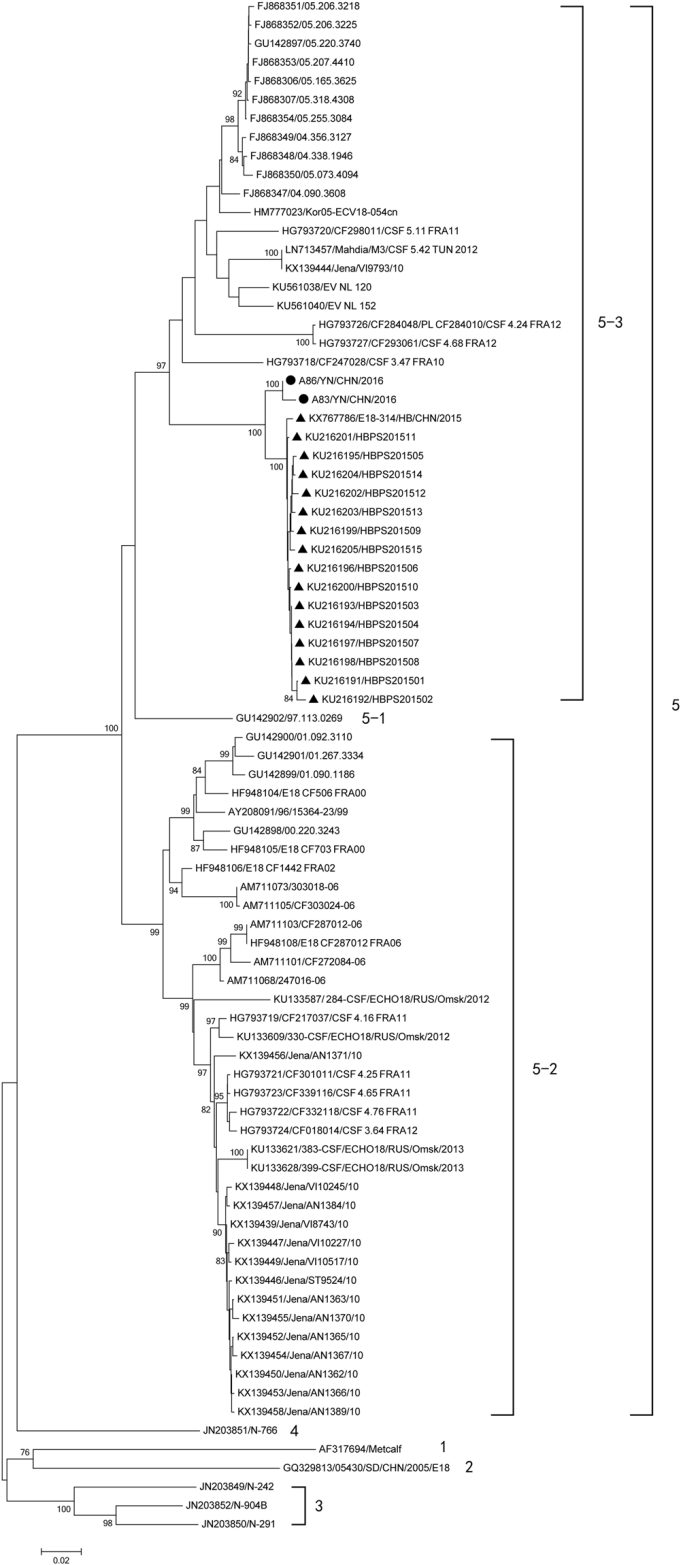
Phylogenetic relationships based on the complete *VP1* sequences of 82 E-18 strains. Two Yunnan E-18 strains and the other 80 E-18 strains available in GenBank database were analysed by nucleotide sequence alignment using the neighbor joining algorithms implemented in the MEGA 6.06 program. Numbers at the nodes indicate bootstrap support for that node (percentage of 1000 bootstrap replicates). The scale bars represent the genetic distance. Only high bootstrap values (>75%) are shown. • Strains isolated in this investigation. ▲ Strains isolated from other provinces of China.

The phylogenetic tree was conducted with 80 strains available in GenBank based on the complete *VP1* sequences (Fig. 1). The 82 strains (including the two Yunnan strains) could be divided into five different genogroups (E-18:1-5). Except for 05430/SD/CHN/2005 (GQ329813), the other Chinese strains clustered together and belonged to genogroup E-18:5, which was also prominent epidemic strains worldwide.

**Figure 2:**
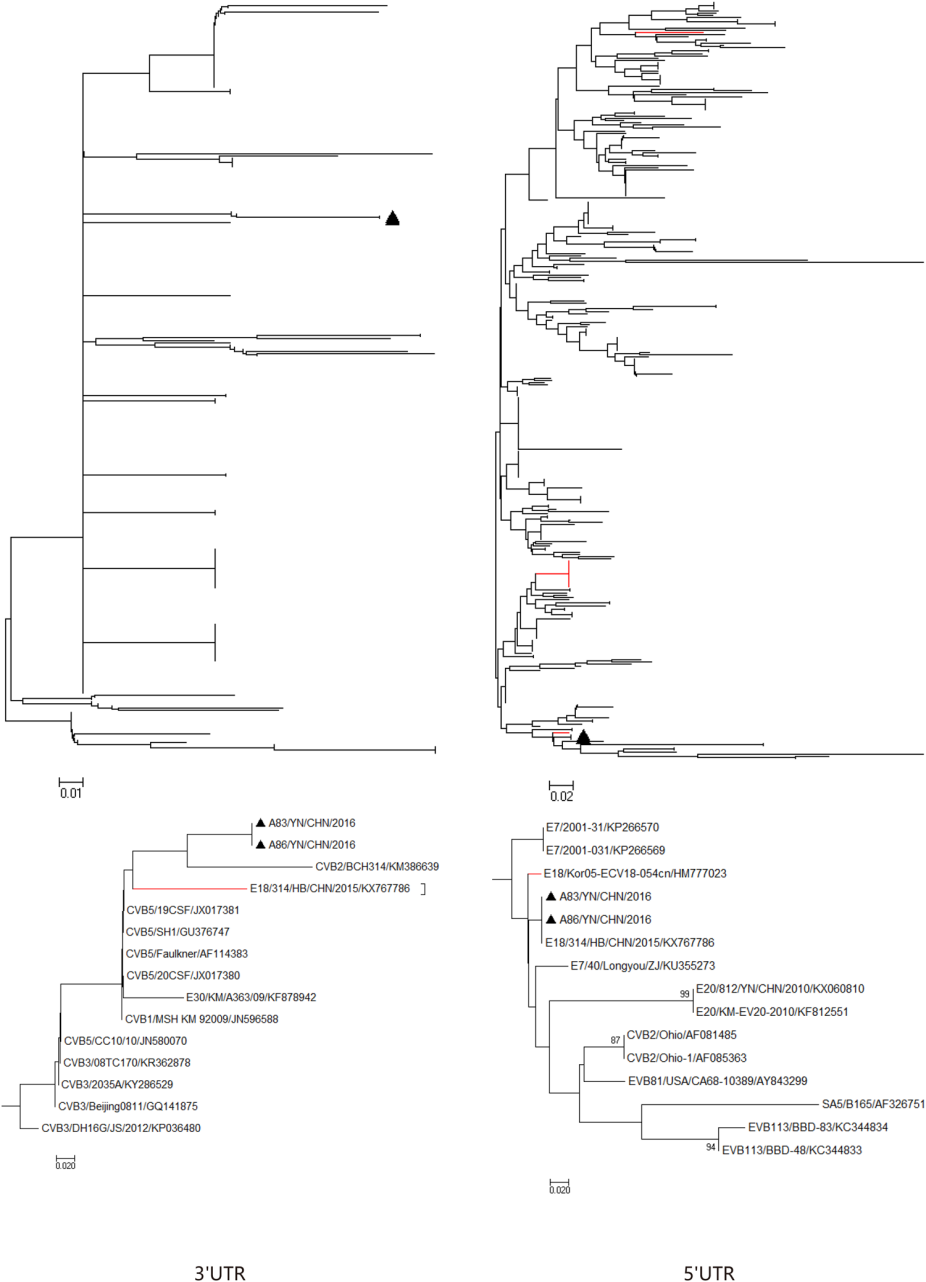
Phylogenetic relationships based on the 5′UTR and 3′UTR sequences of EV-B strains. Two E-18 strains and 343 EV-B strains for 5′UTR, and 349 EV-B strains for 3′UTR available in GenBank database were analysed by nucleotide sequence alignment using the neighbor joining algorithms implemented in the MEGA 6.06 program, respectively. Numbers at the nodes indicate bootstrap support for that node (percentage of 1000 bootstrap replicates). The scale bars represent the genetic distance. Only high bootstrap values (>75%) are shown. Only high bootstrap values (>75%) are shown. ▲ Strains isolated in this investigation. Red denote the other E-18 strains.

**Figure 3:**
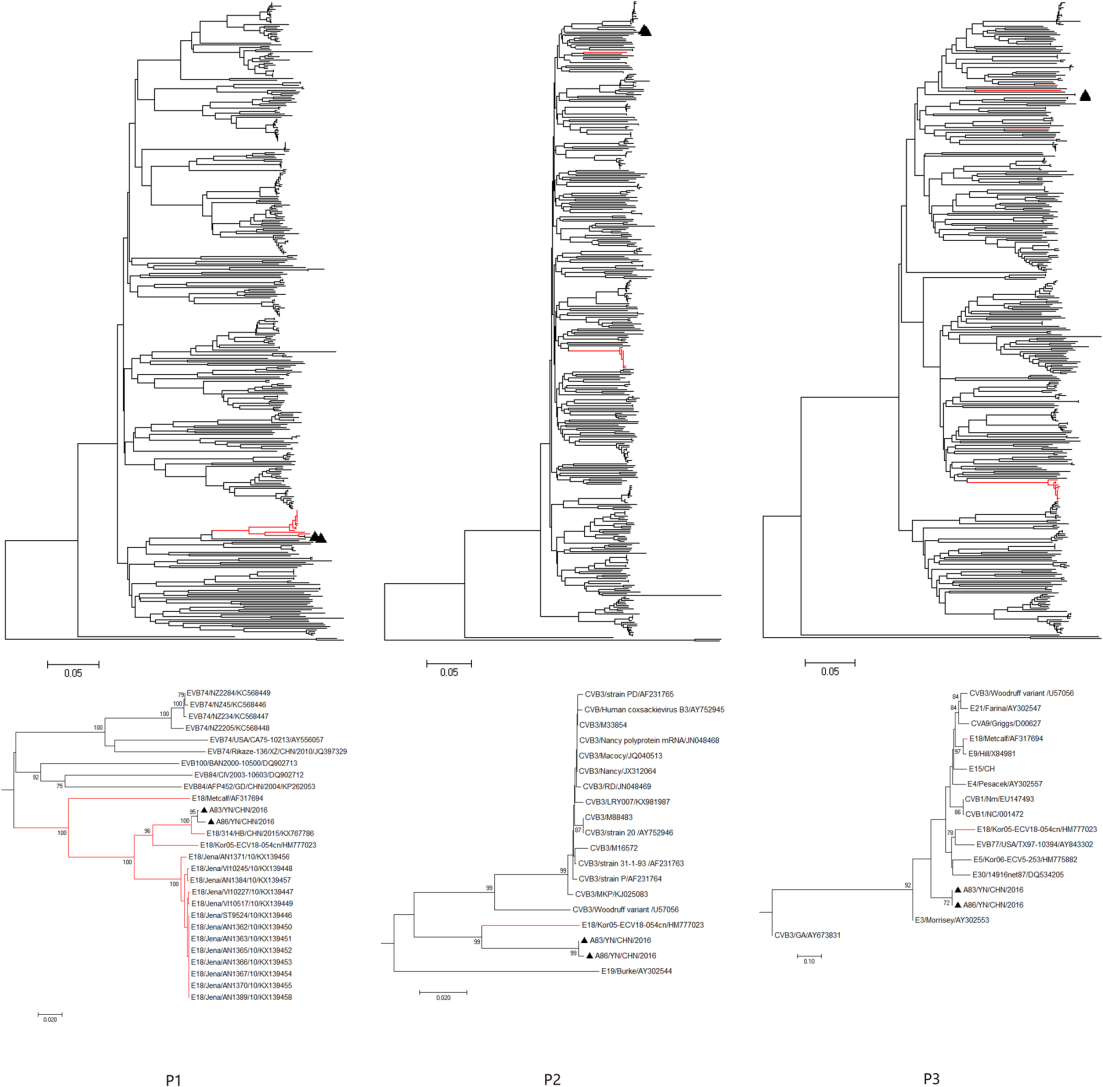
Phylogenetic relationships based on the *P1*, *P2*, and *P3* coding sequences of 379 EV-B strains. Two E-18 strains and 377 EV-B strains available in GenBank database were analysed by nucleotide sequence alignment using the neighbor joining algorithms implemented in the MEGA 6.06 program. Numbers at the nodes indicate bootstrap support for that node (percentage of 1000 bootstrap replicates). The scale bars represent the genetic distance. Only high bootstrap values (>75%) are shown. Only high bootstrap values (>75%) are shown. ▲ Strains isolated in this investigation. Red denote the other E-18 strains.

**Figure 4:**
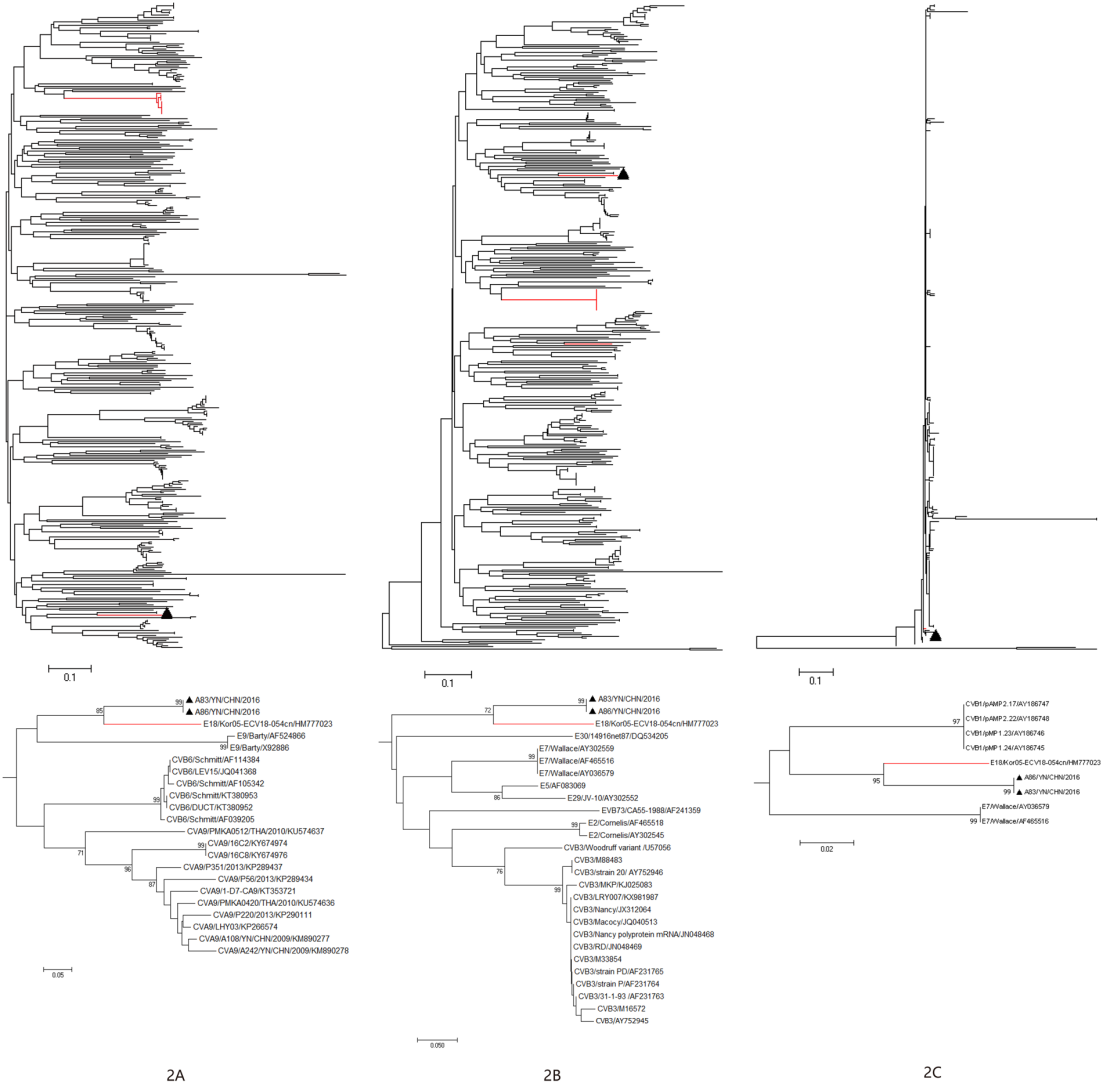
Phylogenetic relationships based on the *2* *A*, *2* *B*, and *2C* coding sequences of 379 EV-B strains. Two E-18 strains and 377 EV-B strains available in GenBank database were analysed by nucleotide sequence alignment using the neighbor joining algorithms implemented in the MEGA 6.06 program. Numbers at the nodes indicate bootstrap support for that node (percentage of 1000 bootstrap replicates). The scale bars represent the genetic distance. Only high bootstrap values (>75%) are shown. Only high bootstrap values (>75%) are shown. ▲ Strains isolated in this investigation. Red denote the other E-18 strains.

**Figure 5:**
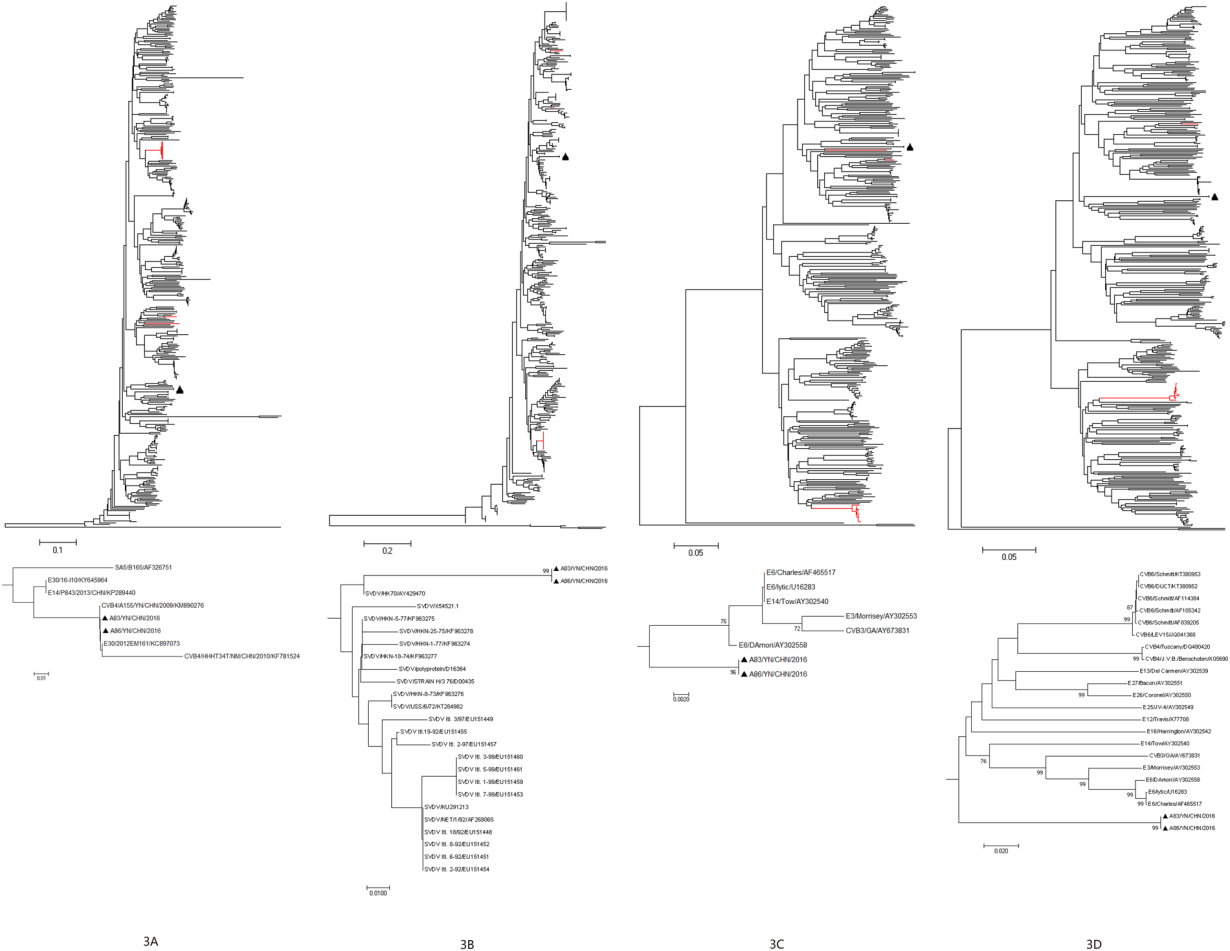
Phylogenetic relationships based on the *3* *A*, *3* *B*, *3C*, and *3D* coding sequences of 379 EV-B strains. Two E-18 strains and 377 EV-B strains available in GenBank database were analysed by nucleotide sequence alignment using the neighbor joining algorithms implemented in the MEGA 6.06 program. Numbers at the nodes indicate bootstrap support for that node (percentage of 1000 bootstrap replicates). The scale bars represent the genetic distance. Only high bootstrap values (>75%) are shown. Only high bootstrap values (>75%) are shown. ▲ Strains isolated in this investigation. Red denote the other E-18 strains.

Phylogenetic analysis of strains A83 and A86 with 377 EV-B strains available in GenBank database were performed based on the other genomic regions respectively (Figs 2–5). In *P1* capsid coding region, the Yunnan E-18 strains only clustered together with other E-18 strains, consistent with the preliminary molecular typing results. However, in the *P3* (or *3A*, *3B*, *3C*  and *3D*) regions (Figs 3 and 5), the two Yunnan strains did not form a lineage with E-18 strains, and grouped together with other EV-B serotypes such as Swine Vesicular disease virus (SVDV) strain HK70. For 3′UTR and *P2* (or *2A* , *2B* and *2C* ) regions (Figs 2, 3 and 4), the two isolates clustered together with one E-18 strain and other EV-B serotypes. Among these, the nt sequence of the *3B* genomic region was the most homologous (93.5%) to the corresponding sequence of SVDV strain HK70. Thus, these results suggest the occurrence of one or more putative recombination events between the two E-18 Yunnan strains and other EV-B serotypes.

In addition, the *VP1* nt and amino acid sequences were compared between the E-18 genogroups (Table 2). The average *VP1* nt sequence divergence between the E-18 genogroups E-18-1, 2, 3, 4, and 5 was 18.6% (range: 15.2–22.1%). However, the divergence was higher than the 14.95% divergence value assigned for EV-A71 subgenotyping^18^. This further confirmed that the E-18 sequences segregated into five distinct genogroups.

**Table 2:** The complete VP1 nucleotide and amino acid sequences comparisons between the E-18 gene clusters.

genotype	A	B	C	D	E
A		79.4	79.6–80.6	78.7	77.9–78.7
B	95.5		82.0–83.0	81.4	79.1–79.8
C	92.3–94.4	94.8–97.2		83.9–84.8	82.7–83.0
D	94.1	96.9	95.6–98.6		83.3–83.6
E	91.3–93.0	92.0–95.5	90.9–96.5	94.1–95.8	

Note: the data in the upper right corner was for nucleotide homology analysis and the data in the lower left corner was for amino acid homology analysis.

### Recombination analysis

**Figure 6:**
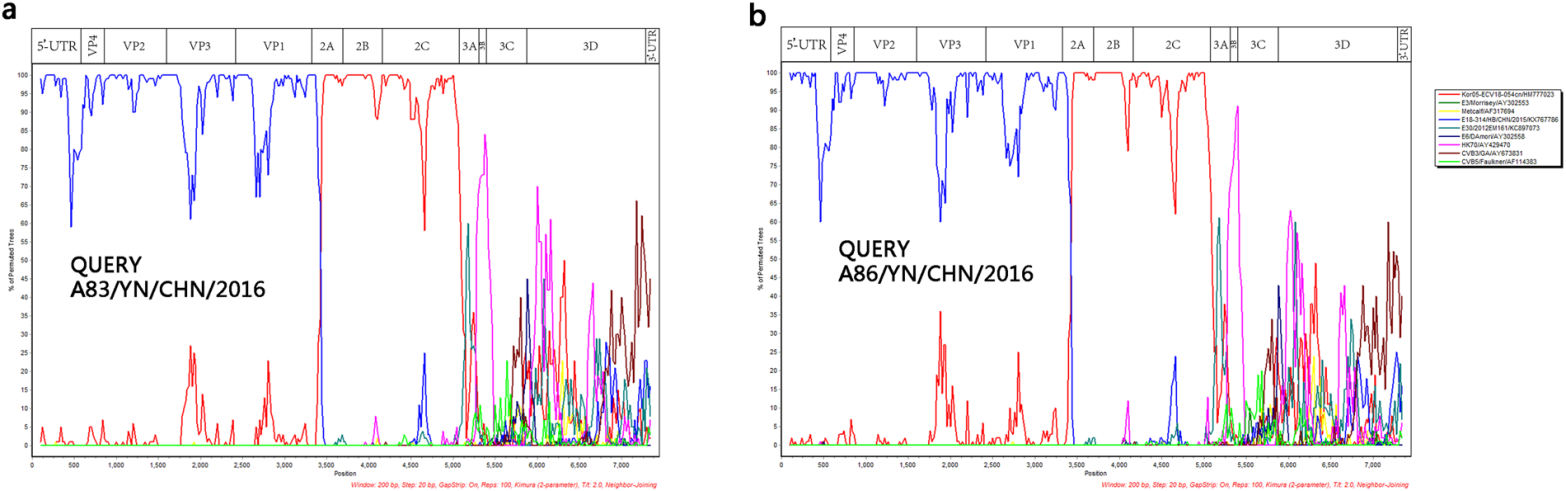
Bootscanning analyses of the complete genome of the two Yunnan E-18 strains. Bootscanning analysis of complete EV-B genomes using a sliding window of 200 nt moving in 20-nt steps. The two Yunnan strains were used as query sequences independently.

To confirm the recombination events between the Yunnan E-18 strains and other EV-B serotypes, bootscanning analyses were performed (Fig. 6). The results also revealed multiple recombination events between the genomic sequence of strains A83 and A86 with other EV-B serotypes, especially, the *3B* region of SVDV strain HK70.

## Discussion

Since 2008, millions of children have developed HFMD each year in mainland China and HFMD has become a serious public health issue. EVs are the causative agents of HFMD. Of these, CV-A16 and EV-A71 are the predominant etiologic agents. Other EV serotypes including CV-A2–6, CV-A8–10, CV-A12, CV-A14, CV-B1–6 and E-4–7, E-9, E-11, E-18, E-25, and E-30 were occasionally detected in sporadic cases or outbreaks of HFMD^19, 20^. Thus, persistent surveillance of HFMD pathogens might help to predict potential predominant serotypes and related disease outbreaks. Recently CV-A6 and CV-A10 have been the prevalent etiologic agents in some provinces of China and in a few countries^19^. Although a vaccine for EV-A71 has been developed, it is important to develop multivalent vaccines against other main serotypes to protect children.

E-18 infection is associated with a variety of clinical presentations from asymptomatic to aseptic meningitis and death^21^. Outbreaks of aseptic meningitis caused by E-18 have been reported in China and worldwide^10–15^. Currently, the E-18 strains available in GenBank were all isolated from aseptic meningitis patients and contact persons. The serotypes of causative agents for aseptic meningitis may overlap with that of HFMD^22^. Thus, the two Yunnan E-18 strains were isolated from HFMD patients, indicating possible connections between E-18 infections and HFMD.

There are currently 80 E-18 complete *VP1* sequences available in the GenBank database, and among these, fifteen strains are full-length genome sequences. The 80 E-18 strains were isolated from different countries, China, India, South Korea, Australia, Netherlands, Germany, Sweden, Russia and France, indicating that E-18 infection is a global distribution. The large divergences between the complete *VP1* coding sequences result in the formation of five clusters in phylogenetic analysis, which indicates that the circulation of distinct E-18 lineages exist in the same and different countries. And the large divergences between Yunnan isolates (A83 and A86) and Hebei strain E18-314/HB/CHN/2015 in non-capsid sequences showed the existence of Chinese E-18 strain apparently evolved along two lineages independently of the other E-18 strains. Additionally, the complete genome sequences of the two strains were highly homologous, indicating that E-18 had been circulating in the environment for an extended period of time.

Recombination is a universal phenomenon for EVs^23, 24^. According to the phylogenetic analyses in different genomic regions, proposed recombination events were observed between the two Yunnan strains and E-30 strain 2012EM161, SVDV strain HK70, E-6 strain D’Amori, and CV-B3 strain GA in corresponding regions, respectively. And bootscanning analyses for the two strains also showed intertypic recombination between E-18, E-30, CV-B3, SVDV and E-6 with the *P3* non-capsid region. Interestingly, the nt sequences of the *3B* genomic region of the two Yunnan strains were the most homologous (93.5%) to the corresponding sequence of the SVDV strain HK70. Whereas, other E-18 strains were no more closely related to HK70 than it was to other CV-B serotypes.

SVD is a highly contagious disease of pigs and is classified as a list A disease by the International Office of Epizootics^25, 26^. Clinically, SVD cannot be distinguished from foot-and-mouth disease (FMD), vesicular stomatitis (VS) and vesicular exanthema of swine (VES), and, therefore, diagnosis depends on laboratory tests. Due to the persistence of the virus in the environment, once the disease spreads, it is very difficult to control. Thus, SVD is notifiable in the world and is strictly controlled by livestock movement. SVDV is the causative agent of SVD^25, 26^. SVDV share a high degree of sequence similarity with CV-B5 in *P1* region and the cross-neutralization was observed between the two viruses^27^. But other genetic regions of SVDV are less homologous to CV-B5. The *P1* protein contains neutralization sites and the (partial) *P1* region-based molecular typing result corresponds well to enterovirus serotypes^28^. At present, the enterovirus typing is generally defined by analysis of the *P1* region. So SVDV is currently classified as a porcine variant of CV-B5^26^. However, it is unclear whether the recombination of SVDV occurred in humans or pigs^29^. In addition, the non-structural protein and un-translated regions, which have diverse functions in the viral replication are characterized by species-specific^11^. Recombination events usually occur in non-structural regions of EV-B^23, 30–32^, which is consistent with our report. E-18 prototype Metcalf strain also is a recombinant with E-9 Hill, which share highly homologous in the *P3* region^33, 34^, and SVDV also is a recombinant with CV-A9 Net/1/63 in the *3D* region. Thus, recombination plays a key role on the evolution and adaptation of the members of EV-B species.

Duo to the number of complete sequences of circulating strains is rather limited, this could result a bias of intra-and inter-typic recombination. In addition, silent transmissions could be the reason of underestimation of recombination events^35^. Thus, although our isolates A83 and A86 share high degree of sequence similarity with SVDV in *3B* region, it is difficult to determine whether the SVDV strain was the donor of Yunnan E-18 strains and more complete genome sequences are required for analysis. In addition, the two Yunnan strains in this study were isolated from patients with HFMD, but we could also not conclude that E-18 was the causative agent of HFMD, for samples not taken from the throat swab, serum and other sites of lesions in the two patients. But after all the two strains were isolated from feces of patients with HFMD. So, further research is needed to elucidate the correlation.

In conclusion, we have reported the full-length genome sequences of two E-18 strains during HFMD surveillance in Yunnan, China. Sequence analysis suggested that the two strains have high genetic diversity compared with the other E-18 strains, intertypic recombination in the non-structural protein region. The high divergence among the E-18 strains reflects that this virus has circulated in the environment for many years and recombination drives the evolution of E-18. This study expands the number of complete genome sequences of E-18 in the GenBank database and provides the molecular epidemiology of E-18 in China, which can help to evaluate the association between E-18 and E-18-related diseases.

## Material and Methods

### Ethics statement

All participants gave written informed consent. The protocol was in accordance with the Helsinki Declaration, and was approved by the Institutional Ethics Boards of the Institute of Medical Biology, Chinese Academy of Medical Sciences & Peking Union Medical College. The human material used were stool samples collected from two patients aged 5 and 4.2 years with HFMD. Stool samples were collected and processed according to standard procedures. Three cell lines, RD, KMB17, and A549 were used to isolate viruses from the stool specimens according to standard procedures^36^. All positive isolates were stored at −80 °C.

### Molecular typing

For molecular serotyping, the viral RNAs were extracted from cell culture supernatants with a QIAamp Viral RNA Mini Kit (QIAGEN, Valencia, CA, USA). Reverse transcription-polymerase chain reaction (RT-PCR) was carried out using a PrimeScript^TM^ One Step RT-PCR Kit Ver.2 (TaKaRa, Dalian, China) with primer pairs 222 and 224^37^. The PCR-positive products were sequenced using an ABI 3730XL automatic sequencer (Applied Biosystems, Foster City, CA, USA) at BGI Sequencing Company (Beijing, China). The partial VP1 sequences were compared with sequences from GenBank using BLAST (http://www.ncbi.nlm.nih.gov/BLAST/).

### Full-length genome amplification

Two long-distance PCR amplifications were performed using a PrimeScript^TM^ One Step RT-PCR Kit Ver.2 (TaKaRa, Dalian, China). The primers used for RT-PCR and sequencing of the full-length genome were designed by a “primer-walking” strategy^38^ and are listed in Table 3. The PCR products were purified using the QIAquick PCR purification kit (Qiagen, Germany), and sequenced in both directions at least two from each strand using ABI 3130 Genetic Analyser (Applied Biosystems, USA).

**Table 3:** Primers used for complete genome amplification and sequencing.

primer	Sequence (5′-3′)	Nucleotide position*	Orientation
224	GCIATGYTIGGIACICAYRT	1977–1996	Forward
222	CICCIGGIGGIAYRWACAT	2969–2951	Reverse
E181F	TTAAAACAGCCTGTGGGTTG	1–20	Forward
E181R	TGTGCCATGAAGGGTGTA	2431–2414	Reverse
E181r	GGAACGCGGTGACTCATC	340–323	Reverse
E181f	CTATAAGGATGCGGCATC	839–856	Forward
E182F	CATAAACGTTAGGGAGA	2714–2730	Forward
E182f	GTACTTCTCGCAGCTGGG	3600–3617	Forward
E184f	CAGAGTGATCAAGAGCA	4263–4269	Forward
E185r	CCCAACTGGGATGTACAT	5749–5732	Reverse
E186r	CCTGGGTTTTGGTGAAAG	6520–6503	Reverse
E187f	GACAAGGGAGAGTGTTT	7018–7034	Forward
E188R	ACCGAATGCGGAGAATTTAC	7410–7391	Reverse

*Numbering according to the genome of E-18 strain E18-314/HB/CHN/2015.

### Sequence analysis and recombination analysis

Nucleotide and amino acid sequence alignment were performed by using Geneious (5.6.5)^16^. Phylogenetic analysis was conducted via MEGA version 6.06 using the neighbour-joining method with 1000 duplicates and bootstrap values greater than 75% were considered statistically significant. Bootscanning analysis were performed using the Simplot 3.5.1 program with a 200-nt window moving in 20 nt steps^38^.

### Nucleotide sequence accession number

The complete genome sequences of the E-18 strains A83/YN/CHN/2016 and A86/YN/CHN/2016 described in this study were deposited in the GenBank database under the accession number: KY828851-KY828852.
